# Evaluation of the depth of sedation in an intensive care unit based on the photo motor reflex variations measured by video pupillometry

**DOI:** 10.1186/2110-5820-3-5

**Published:** 2013-02-22

**Authors:** Ouri Rouche, Aurore Wolak-Thierry, Quentin Destoop, Lucas Milloncourt, Thierry Floch, Pascal Raclot, Damien Jolly, Joël Cousson

**Affiliations:** 1 Service de Réanimation Polyvalente Hôpital Robert Debré, Reims Cedex, CHU 51092, France; 2 Unité d'analyse méthodologique, Reims Cedex, CHU 51092, France

**Keywords:** Sedation, Photomotor reflex, Video pupillometry, Bispectral index

## Abstract

**Background:**

Evaluating depth of sedation in the intensive care unit (ICU) is crucial for the management of mechanically ventilated patients but can be challenging in some situations. Because the depth of hypnosis is correlated with the decrease in photomotor reflex (PMR), we suggest using pupillometric video as an automated, noninvasive, simple, and reproducible technique to evaluate the depth of sedation in ICU patients. We compare the effectiveness of this procedure to the bispectral index (BIS).

**Methods:**

Thirty-one patients requiring sedation and ventilation were included in this monocentric, observational study. The posology of hypnotics and morphinics were based on the Richmond Agitation and Sedation Scale (RASS). PMR was measured by the Neurolight® (IDMED) system and BIS value by BIS Vista® (Anandic Medical Systems). RASS, PMR, and BIS were measured three times daily in all patients. Data acquired by pupillometric video included variation in pupillary diameter (ΔPD), latency time (LT), and maximal speed of pupillary constriction (Vmax). These parameters were analyzed after having classified BIS values in three groups (<40 heavy sedation; 40 ≤ BIS ≤ 60 acceptable sedation; >60 light sedation). Exclusion criteria were neurological or ophthalmologic pathologies that could interfere with PMR.

**Results:**

There was a significant difference in Vmax and ΔPD between the BIS < 40 group and 40 ≤ BIS ≤ 60 groups (*p* < 0.0001 for each) and between the BIS < 40 and BIS > 60 groups (*p* < 0.0001 for each). There were no significant differences in Vmax and ΔPD between the 40 ≤ BIS ≤ 60 and BIS > 60 groups. There was no correlation between any of the BIS groups and LT.

**Conclusions:**

Vmax and ΔPD seem to be relevant criteria compared with the BIS and the RASS. Pupillometric video monitoring of depth of sedation could be beneficial in ICU patients, especially for those under myorelaxant drugs, where no clinical evaluation of sedation is possible.

## Background

Large numbers of patients admitted to the intensive care unit (ICU) are sedated when mechanically ventilated [1]. Evaluating the depth of sedation is a crucial element in the management of these patients. Indeed, when sedation is too deep, it can lead to hemodynamic instability, and when too light, it can cause ventilatory difficulties, accidental extubation, or memorization phenomena. Assessing depth of sedation may be difficult in some situations. For example, in patients with acute respiratory distress syndrome (ARDS), ventilation often is difficult and may require myorelaxant drugs, which make it impossible to evaluate clinically the depth of sedation. In these cases, current practice suggests shortly interrupting the administration of these drugs on a daily basis to evaluate correctly the sedation level of the patient.

To optimize the quality of sedation, several review articles and guidelines have been published that outline recommended management [2-4]. Because the depth of hypnosis is known to be correlated with a decrease in photomotor reflex (PMR) [5-10], it could be beneficial to develop an automated, noninvasive, simple, and reproducible technique to evaluate efficiently the depth of sedation in an ICU based on the pupillary reflex.

Depth of sedation is not commonly monitored by the bispectral index (BIS) in the ICU setting; its use is controversial. Several studies have attempted to validate this device compared with commonly used sedation scales. BIS values suffer from a wide dispersion of values compared with predefined clinical scale scores. The main challenge is the overestimation of BIS values due to electromyographic activity [11]. In deeply sedated patients, values remain widely dispersed but are correlated to clinical sedation scale values (e.g., RASS −5).

The objective of this monocentric observational study was to evaluate the effectiveness of pupillometric video using the Neurolight® (IDmed™) system compared with the Richmond Agitation and Sedation Scale (RASS) [12-14] and the bispectral index (BIS).

## Methods

### Study population

Patients hospitalized in the ICU of Reims Champagne-Ardennes University Hospital between 1 June and 31 August 2011 were eligible for inclusion in this study. The ICU has a capacity of 15 beds with a nurse to patient ratio of 1:3. All ICU patients who required heavy sedation to be adapted optimally to mechanical ventilation were included in the study. The study protocol was approved by the ethics committee of our institution. Exclusion criteria included all intrinsic or extrinsic patient factors that could interfere with PMR values, namely: i) use of any of the following drugs: clonidine, tramadol, droperidol, metoclopramide, ketamine, adrenalin, or calcium channel blockers [8,15,16]; ii) neurological pathologies (severe head trauma, subarachnoid hemorrhage, stroke, intracerebral hemorrhage, or multiple sclerosis); and iii) ophthalmological pathologies (cataract, conjunctivitis, or high myopia).

### Data recorded

The doses of midazolam and sufentanil were based on the RASS, with a target score between RASS −4 and −5. These drugs, which are commonly used in the ICU, do not alter the PMR [6,10].

PMR was measured with a 320 Lux flash of light using the Neurolight**®** (IDmed, Marseille, France). Three measurements per day were taken for a 48-hour period. BIS values also were recorded (BIS Vista®, Anandic Medical Systems, Feuerthalen, Switzerland). The data recorded by the pupillometric video comprised: variation of pupillary diameter (ΔPD), latency time (LT), and maximal speed of pupillary constriction (Vmax). These measurements were analyzed after classification of BIS values into three groups (<40 heavy sedation; 40 ≤ BIS ≤ 60 acceptable sedation; >60 light sedation) [17]. All assessments of PMR, BIS, and RASS followed a standardized protocol for the study. Each patient had three evaluations per day, at predefined hours and under equivalent lighting conditions. The same physician performed all measurements (O.R.).

### Number of measures needed

#### Statistical analysis

Quantitative variables are presented as mean ± standard deviation (SD) and qualitative variables as number and percentage. After having verified that the distribution was normal, we conducted repeated measures analysis of variance (ANOVA) for the variables of interest (i.e., ΔPD, LT, Vmax). If repeated measures ANOVA was significant, we considered each measure independently and performed one-way ANOVA adjusted for the subject, day, and measure to compare pupillometry parameters between the three BIS groups. When a significant difference was found, two by two comparisons between the three groups of BIS was performed using Bonferroni tests.

For all analysis, *p* < 0.05 was considered statistically significant. Analyses were performed using SAS version 9.3 (SAS Institute Inc., Cary, NC).

## Results

A total of 167 measures of PMR and BIS were conducted on 31 patients. The patient characteristics are summarized in Table 1. The medication taken by the patients during the study period is detailed in Table 2. There were no significant differences between groups in LT. Repeated measures ANOVA showed significant differences for Vmax and ΔPD (*p* < 0.0001). Subsequent two by two comparison using Bonferroni tests revealed significant differences between the groups BIS < 40 and 40 ≤ BIS ≤ 60, and between the BIS < 40 and BIS > 60 groups for Vmax and ΔPD (*p* < 0.0001 for all comparisons; Table 3).

**Table 1:** Baseline characteristics of the study population

**Variable**	**All patients (n = 31)**
Age (yr)	57 ± 14
Weight (kg)	81.7 ± 18.4
Height (cm)	166 ± 32
SAPS II	56.3 ± 15.8
PaO_2_/FiO_2_ (mmHg)	194 ± 114
RASS score	−4.45 ± 0.71
Male sex	23 (74.2)
Myorelaxant drugs	20 (64.5)
Death in ICU	20 (64.5)
Indications for ICU entry	
Septic shock	20 (64.5)
Cardio circulatory arrest	8 (25.8)
Status epilepticus	1 (3.23)
Acute severe asthma	1 (3.23)
Coma	1 (3.23)

Kg, kilograms; cm, centimeters; SAPS II, Simplified Acute Physiology Score; PaO_2_, partial pressure of oxygen in arterial blood; FiO_2_, fraction of inspired oxygen; ICU, intensive care unit.

**Table 2:** Average doses used for sedation and medications taken by the study population during the study period

**Sedation**	**N**	**Mean (SD)**
Pentothal (mg/h)	1	350.00
Sufentanil (gamma/h)	31	20.00 (5.24)
Midazolam (mg/h)	31	11.61 (4.72)
Medication	N	%
Norepinephrine	26	83.87
Enoxaparin	14	45.16
Gentamicin	12	38.71
Ceftriaxone	10	32.26
Aspirin	10	32.26
Atorvastatin	8	25.81
Fondaparinux	6	19.35
Amiodarone	6	19.35
Dobutamine	6	19.35
Ticarcillin/clavulanic acid	5	16.13
Clopidogrel	5	16.13
Furosemide	4	12.90
Piperacillin	3	9.68
Metronidazole	2	6.45
Ceftazidime	1	3.23
Levofloxacin	1	3.23
Imipenem - cilastatin sodium	1	3.23
Vancomycin	1	3.23

SD, standard deviation.

**Table 3:** **Adjusted means of pupillometric measures (Vmax, LT, and** Δ**PD) for each BIS group**

	**BIS**		
Pupillometric	BIS < 40	40 ≤ BIS ≤ 60	BIS > 60
Measures	(N = 68)	(N = 62)	(N = 37)
Vmax (mm/s)	1.14	1.35	1.40
LT (ms)	259.25	237.92	228.00
ΔPD (mm)	0.3	0.4	0.43

*p* < 0.0001 for Vmax and ΔPD between BIS < 40 and 40 ≤ BIS ≤ 60 groups.

*p* < 0.0001 for Vmax and ΔPD between BIS < 40 and BIS > 60 groups.

## Discussion

Our results show that depth of sedation seems to be efficiently evaluated by the PMR measured by the Neurolight®. The PMR was correlated to the RASS objectives. Compared with the BIS groups, a significant difference was found between the groups BIS < 40 and 40 ≤ BIS ≤ 60, and between the BIS < 40 and BIS > 60. We could not show a difference between the groups 40 ≤ BIS ≤ 60 and BIS > 60, probably because of the limitations of the BIS use in ICU and perhaps because we did not have enough measurements.

Other studies showed similar interest in the PMR for the evaluation of the sedation. These studies did not concern ICU patients; however, they all showed that the LT variable was probably of no interest in the evaluation of sedation [18,19].

This device appears to be of utility in the ICU, where patients can often be treated by myorelaxant drugs (muscle-blocking drugs do not alter the PMR [20]), because no clinical scale can be used in this situation. The utility of the BIS in the ICU remains to be defined [21], but it is undoubtedly still useful when clinical scales are ineffective, especially when muscular relaxant drugs are used [21-28]. Actually, there is no “gold standard” for evaluating the sedation status in patients under myorelaxant drugs.

Current guidelines recommend interruption of myorelaxant infusion and clinical evaluation of the sedation level when the muscle blockade has been reversed. However, these guidelines can sometimes be difficult to implement, due to the very unstable status of this category of critically ill patients.

The Neurolight® manufacturer (IDmed™) gives an indication of the pupil reactivity (very good, good, week, null) using all three variables measured by the device. To the best of our knowledge, no thresholds have been defined for pupillometry through a large-scale, randomized, controlled trial yet. We only considered the raw results of Vmax, ΔPD, and LT, and therefore, did not take into consideration the pupillary reactivity indicator calculated by the Neurolight®.

## Conclusions

Vmax and ΔPD seem to be useful for the evaluation of the sedation in ICU compared with the BIS. This noninvasive technique of monitoring the depth of sedation can be highly beneficial especially for patients under myorelaxant drugs due to the impossibility of using any clinical sedation scales and the fact that these drugs do not alter PMR. This equipment could help reduce the risk of overdose or under dose of hypnotics in the ICU. A larger study is necessary to confirm these results and define cutoff values.

## Competing interests

The authors declare that they have no competing interests.

## Authors’ contributions

OR Carried out the measurements and wrote the article. AWT Carried out all the statistical paragraph and corrected the article. QD Helped including patients in the study and corrected the article. LM Helped including patients in the study and corrected the article. TF Carried out the study plan and corrected the article. PR Carried out the study plan and corrected the article. DJ Carried out all the statistical part of the study and corrected the article. JC Suppervised the study, carried out the study plan and corrected the article. All authors read and approved the final manuscript.
